# Symptoms, SARS-CoV-2 Antibodies, and Neutralization Capacity in a Cross Sectional-Population of German Children

**DOI:** 10.3389/fped.2021.678937

**Published:** 2021-10-04

**Authors:** Otto Laub, Georg Leipold, Antoaneta A. Toncheva, David Peterhoff, Sebastian Einhauser, Patrick Neckermann, Natascha Borchers, Elisangela Santos-Valente, Parastoo Kheiroddin, Heike Buntrock-Döpke, Sarah Laub, Patricia Schöberl, Andrea Schweiger-Kabesch, Dominik Ewald, Michael Horn, Jakob Niggel, Andreas Ambrosch, Klaus Überla, Stephan Gerling, Susanne Brandstetter, Ralf Wagner, Michael Kabesch, Corona Virus Antibodies in Children from Bavaria (CoKiBa) Study Group, Bettina Aichholzer

**Affiliations:** ^1^Pediatric Office Laub, Rosenheim, Germany; ^2^Pediatric Office Dr. Leipold, Regensburg, Germany; ^3^University Children's Hospital Regensburg (KUNO) at the Hospital St. Hedwig of the Order of St. John, University of Regensburg, Regensburg, Germany; ^4^Institute of Medical Microbiology and Hygiene, Molecular Microbiology (Virology), University of Regensburg, Regensburg, Germany; ^5^Institute of Clinical Microbiology and Hygiene, University Hospital, Regensburg, Germany; ^6^Member of the Research and Development Campus Regensburg (WECARE) at the Hospital St. Hedwig of the Order of St. John, Regensburg, Germany; ^7^Pediatric Office Dr. Heuschmann & Dr. Ewald, Regenstauf, Germany; ^8^Pediatric Office Dr. Horn, Schönau, Germany; ^9^Maganamed Limited, Regensburg, Germany; ^10^Institute of Laboratory Medicine, Microbiology and Hygiene, Hospital of the Order of St. John, Regensburg, Germany; ^11^Institute of Clinical and Molecular Virology, FAU Erlangen-Nuremberg and Universitätsklinikum Erlangen, Erlangen, Germany

**Keywords:** antibody, neutralizing, COVID-19, SARS-CoV-2, children

## Abstract

**Background:** Children and youth are affected rather mildly in the acute phase of COVID-19 and thus, SARS-CoV-2 infection infection may easily be overlooked. In the light of current discussions on the vaccinations of children it seems necessary to better identify children who are immune against SARS-CoV-2 due to a previous infection and to better understand COVID-19 related immune reactions in children.

**Methods:** In a cross-sectional design, children aged 1–17 were recruited through primary care pediatricians for the study (a) randomly, if they had an appointment for a regular health check-up or (b) if parents and children volunteered and actively wanted to participate in the study. Symptoms were recorded and two antibody tests were performed in parallel directed against S (in house test) and N (Roche Elecsys) viral proteins. In children with antibody response in either test, neutralization activity was determined.

**Results:** We identified antibodies against SARS-CoV-2 in 162 of 2,832 eligible children (5.7%) between end of May and end of July 2020 in three, in part strongly affected regions of Bavaria in the first wave of the pandemic. Approximately 60% of antibody positive children (*n* = 97) showed high levels (>97th percentile) of antibodies against N-protein, and for the S-protein, similar results were found. Sufficient neutralizing activity was detected for only 135 antibody positive children (86%), irrespective of age and sex. Initial COVID-19 symptoms were unspecific in children except for the loss of smell and taste and unrelated to antibody responses or neutralization capacity. Approximately 30% of PCR positive children did not show seroconversion in our small subsample in which PCR tests were performed.

**Conclusions:** Symptoms of SARS-CoV-2 infections are unspecific in children and antibody responses show a dichotomous structure with strong responses in many and no detectable antibodies in PCR positive children and missing neutralization activity in a relevant proportion of the young population.

## Introduction

Early in the COVID-19 pandemic, children and adolescents were thought to be important transmitters of the disease but were also believed to be only mildly affected (1). Later, evidence increased that children are not major spreaders (2–4). However, a pediatric multiorgan immune syndrome in children and youths was reported (5), occurring weeks to months after the SARS-CoV-2 infection, also in children with mild or no symptoms in the initial phase of the disease. Recent studies linked PIMS to the presence of antibodies to SARS-CoV-2 and some authors suggested that high levels of antibodies against SARS-CoV-2 may in fact contribute to the occurrence of the full-fledged syndrome (6). These observations indicate that immune reactions to SARS-CoV-2 exposure may differ, at least in strength, between children and adults.

When vaccination for SARS-CoV-2 was first administered to adults, stronger systemic vaccination reactions to the vaccine were reported in younger individuals (7). In some of our cases, high antibody levels were already observed directly after vaccination when these symptoms occurred (own observation), suggesting a possibility that these individuals may have had an unnoticed SARS-CoV-2 infection previously. With vaccination of children against SARS-CoV-2 in sight, it is important to better identify those that were already infected and to improve our understanding of SARS-CoV-2 related immune responses in children overall.

In many children allegedly mild or inapparent infections occurred and PCR testing was performed rarely. Therefore, we screened a large number of children in rather severely affected areas of Bavaria (Southern Germany) for symptoms as well as overall and neutralizing antibody levels against SARS-CoV-2 in the first pandemic wave in spring of 2020, in a population-based approach.

## Methods

### Study Design and Population

**Figure 1:**
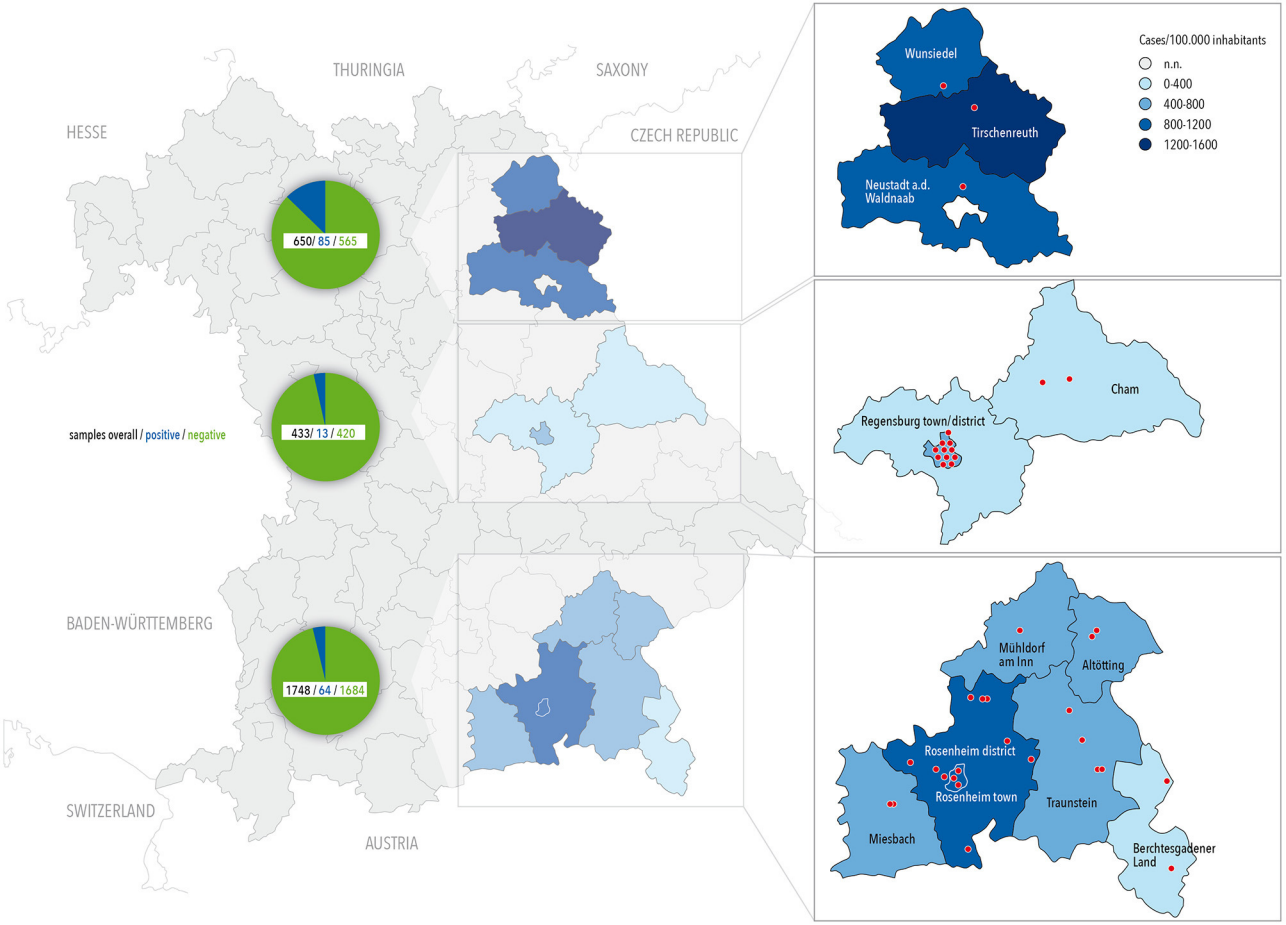
Map of Bavaria with location of centers contributing to the survey (red dots) and COVID-19 prevalence until July 2020 (color coded by county). Number for overall, negatively and positively tested children are given in the circle chart.

In a cross-sectional design we investigated children from three distinct regions of South East Germany to assess the true prevalence of SARS-CoV-2 infections in areas with very differently reported infection rates by antibody testing. We established a network of pediatricians who volunteered to take part in the study and focused on three areas/counties within Bavaria with very high, moderate, and average infection rates as indicated by positive PCR tests per 100,000 inhabitants according to the Robert Koch Institute, the German center for disease prevention (Figure 1). The assessment and sample collection took place in three study areas: Tirschenreuth; Regensburg city and county; and Oberbayern/ alpine region from May 22nd to July 22nd, 2020. In areas where the number of willful study participants exceeded the capacity of local pediatricians, a study team supported sample collections.

Invitation to participate for children aged 1–14 years was based on two approaches: (a) All children of that age group who were scheduled for a prevention program visit in 2020 with the respective pediatrician were invited to participate (random selection) and (b) all children of families who actively wanted to participate were also tested (own intention to participate). In approach (b), also siblings older than 14 years were allowed to participate in the study, as for ethical reasons, children older than 14 could not be excluded from antibody testing if families presented them together with younger siblings for testing. The study was approved by the Ethics Committee of the University of Regensburg (file-number: 20-1865-101).

### Data Collection and Management

All data were collected in an online survey using self-administered parental questionnaires. The questionnaires can be obtained upon request from the authors. All acquired data was fully anonymized and only accessible at an individual level to the participant using an individual code on the Qnome platform (www.qnome.eu) as previously described in detail (8). Clinical data was entered by the parents in an online survey. That way, anonymization of data on the level of the dataset was achieved while the test values were directly accessible to parents.

### SARS-CoV-2 Antibody Tests

Blood was taken from all participants by venipuncture. Specific antibody response to SARS-CoV-2 was evaluated by the use of two different test kits: the commercially available, licensed qualitative Elecsys Anti-SARS-CoV-2 (Roche Diagnostics, Rotkreuz, Switzerland; https://diagnostics.roche.com) with a sensitivity of 99.5% and a specificity of 99.8%, according to the manufacturer; and a validated and published in-house ELISA with a sensitivity of 96% and a specificity of 99.3% as previously reported (9). The Elecsys Anti-SARS-CoV-2 assay does not discriminate between the antibody type(s) present and can detect IgA, IgM, and IgG. The test is based on a recombinant nucleocapsid (N) antigen and has a cutoff value of 1.0 (S/Co). The in-house ELISA is based on SARS-CoV-2 S-protein's receptor-binding domain, quantifies total IgG and has a cutoff value of 1.0 (S/Co). The detected reactivity correlates with the SARS-CoV-2 neutralization titer as described previously (9). All samples with S/Co <1.0 were considered negative.

### SARS-CoV-2 Neutralization Test

Neutralizing antibodies were evaluated by titration of sera against SARS-CoV-2 pseudotyped Vesicular Stomatitis Virus (VSV). The test is based on VSV-ΔG^*^FLuc pseudotyped with SARS-CoV-2-Spike-ΔER, which correlates with SARS-CoV-2 neutralization as described previously (10, 11). Pseudoviral titers were determined by limited dilution and fluorescence microscopy. For all samples, a fixed inoculum of 25,000 ffu was neutralized for 1 h and luciferase activity was determined 20 h post infection of HEK293T-ACE2^+^-cells. IC50 values were fitted using the algorithm: ‘log (inhibitor) vs. normalized response'. Data were analyzed and Spearman's correlations (R) were calculated in GraphPad Prism 8 software (GraphPad Software, San Diego, USA).

### Statistical Analyses

Descriptive statistics were calculated using frequencies (percentages) for categorical data and median (interquartile range) for metric data. Participants' characteristics and symptoms are presented stratified by antibody response. Differences between groups were analyzed using χ^2^-tests for categorical variables and *t*-test for independent groups, respectively. All analyses were performed using SPSS.23.

## Results

Overall, 2,934 children participated in the study of whom 2,906 were tested successfully with at least one of the two applied antibody tests and 2,832 (96.5%) had also entered necessary study data in the online tool. Demographic data of the children participating in the study are given in Table 1 and locations of test-centers across counties are depicted in Figure 1.

**Table 1:** Characteristics of study participants stratified for antibody (AB) test result.

**General characteristics**	**Negative AB test (*N* = 2,670)**	**Positive AB test (*N* = 162)**	***p***
Study participation due to…			
random selection (health check-up), % (*N*)	66.0 (1,763)	32.1 (52)	
own intention to participate, % (*N*)	34.0 (907)	67.9 (110)	<0.001^*^
Sex (male), % (*N*)	51.7 (1,380)	50.6 (82)	0.792
Age (years) (Md, IQR)	7 (4.0–10.0)	8 (4.7–11.0)	0.070
	(range 0–17)	(range 0–16)	
Any chronic disease, % (*N*)	12.3 (329)	9.3 (15)	0.247
Does your child usually attend…			
Nursery, % (*N*)	6.1 (163)	4.9 (8)	
Kindergarten, % (*N*)	27.5 (733)	23.5 (38)	
Elementary school, % (*N*)	30.3 (809)	29.0 (47)	
Secondary school (Mittelschule), % (*N*)	4.9 (130)	9.9 (16)	
Secondary school (Realschule), % (*N*)	8.5 (227)	11.1 (18)	
Grammar school, % (*N*)	11.0 (295)	9.9 (16)	
School for special needs, % (*N*)	0.6 (17)	1.2 (2)	
None of them, % (*N*)	11.1 (296)	10.5 (17)	0.138
SARS-CoV-2 PCR testing, % (*N*)	8.8 (234)	17.9 (29)	<.001^*^
Positive SARS-CoV-2 PCR test, % (*N*)	0.2 (6)	9.3 (15)	<0.001^*^
Hospitalization due to COVID-19, % (*N*)	0.2 (6)	1.2 (2)	0.019^*^
Household member COVID-19, % (*N*)	6.0 (161)	47.5 (77)	<0.001^*^
Any symptom, % (*N*)	70.1 (1,871)	76.5 (124)	0.080

* *p < 0.05; chi^2^ test, t-test for independent groups. IQR, interquartile range. Md, median*.

**Figure 2:**
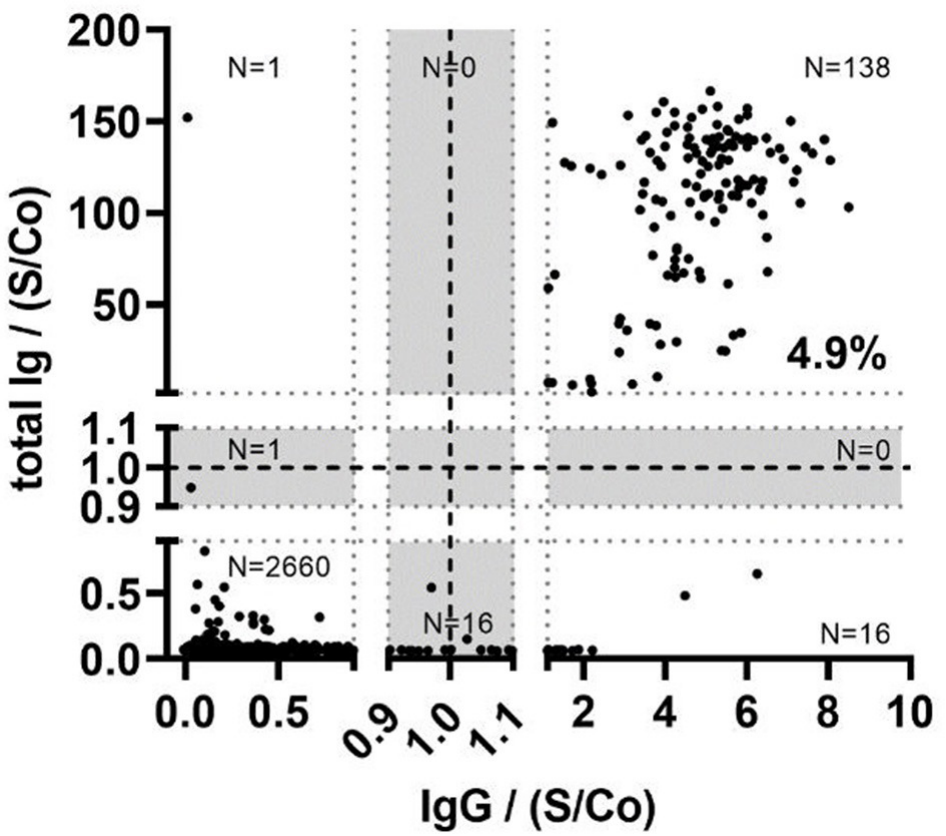
Comparison between the N protein directed Elecsys Anti-SARS-CoV-2 assay (total Ig) and the S protein directed in-house SARS-CoV-2 assay detecting IgG (IgG) in the total study population (*N* = 2,832). Strong dotted lines represent the assay cutoff values, ±10% borderline intervals (gray areas). Signal-to-cutoff (S/Co) ratios are given for both assays.

Overall, 161 participants were classified seropositive with any test^*^, of which 158 were ELISA positive and 139 showed a positive ELECSYS signal, yielding a total concordance of 83.9 % (*n* = 135 positive in both tests) and a total discordance of 16.1 % (*n* = 23 ELECSYS-positive/ELISA-negative; *n* = 3 ELECSYS-positive/ELISA) (Figure 2). A positive result in at least one of the two tests defined a positive case.

Strong regional differences were observed in the prevalence of SARS-CoV-2 antibodies in children (Figure 1). Overall, children in the heavily affected county of Tirschenreuth (with 1,638 positive PCR tests/100,000 inhabitants when the survey was performed) had positive antibody response 3–4 times more often than in the two other test regions, with 586 positive PCR tests/100,000 inhabitants in Regensburg and 1,111 positive PCR tests/100,000 inhabitants in Rosenheim (September 2020). When only those children randomly selected [approach (a)] and only one child (the youngest) per family were included in the analysis, 7.2% of tested children where positive in Tirschenreuth, 3.1% in Regensburg and 1.8% in Oberbayern/Alpine region. In those who participated on their own intention, e.g., due to symptoms that may have been related to COVID-19 or suspected contact to a COVID-19 patient [approach (b)], 15.9% were found positive in Tirschenreuth, 2.3% in Regensburg and 7.8% in Oberbayern/Alpine region, again taking only one child per family into account.

The older the children, the more positive SARS-CoV-2 tests were found, with 4.9% positive in the 0–6 year-olds (*n* = 1,299), 5.7% in the 7–10 year-olds (*n* = 849) and 7.3% positive in the 11–17 year-olds (*n* = 684). Children with chronic diseases tended to be slightly less often positive (4.3% of 344) than those without chronic diseases (5.9% of 2,488). Within the study population, only 263 children had already received a SARS-CoV-2 PCR test previously and 21 had a positive test result. Of these, 15 individuals showed elevated antibody responses (71.4%) while in 6 subjects no antibody response in any of the two tests could be found. Two hundred and thirty-eight children lived in a household with a positively tested family member and of these, 32.4% developed antibodies against SARS-CoV-2. Thus, living with a SARS-CoV-2 positive family member is the single most prominent association with a SARS-CoV-2 infection in children in our study population. We assessed symptoms potentially related with SARS-CoV-2 infections in our study population but found very few specific features (other than the loss of smell and taste) which would allow to discriminate COVID-19 from common viral infections in children (Table 2).

**Table 2:** Symptoms of study participants after antibody measurement: stratified for antibody (AB) test result.

**Symptoms**	**Negative AB test (*N* = 2,670)**	**Positive AB test (*N* = 162)**	***p***
No symptoms, % (*N*)	30.1 (804)	23.5 (38)	0.072
Runny nose, % (*N*)	42.5 (1,135)	32.7 (53)	0.014^*^
Sore throat, % (*N*)	28.2 (753)	18.5 (30)	0.007^*^
Headache, % (*N*)	24.3 (648)	24.1 (39)	0.955
Dizziness, % (*N*)	6.5 (173)	4.9 (8)	0.436
Exhaustion/ fatigue, % (*N*)	24.0 (640)	25.3 (41)	0.699
Muscle aches, % (*N*)	14.0 (373)	16.0 (26)	0.460
Inflammation of the eyes, % (*N*)	4.4 (117)	3.1 (5)	0.430
Loss of smell, % (*N*)	1.0 (27)	4.9 (8)	<0.001^*^
Loss of taste, % (*N*)	2.4 (64)	6.8 (11)	0.001^*^
Shortness of breath, % (*N*)	5.1 (137)	3.7 (6)	0.420
Coughing, % (*N*)	41.0 (1,096)	30.9 (50)	0.010^*^
Fever, % (*N*)	37.6 (1,004)	38.3 (62)	0.865
Chills, % (*N*)	7.3 (194)	3.7 (6)	0.086
Rash, % (*N*)	5.3 (142)	2.5 (4)	0.111
Diarrhea, % (*N*)	16.5 (441)	13.0 (21)	0.235
Nausea, % (*N*)	11.4 (304)	9.9 (16)	0.556
Loss of appetite/difficulty feeding, % (*N*)	11.2 (298)	5.6 (9)	0.026^*^
Other symptoms, % (*N*)	2.5 (66)	2.5 (4)	0.998

* *p < 0.05; chi^2^ test*.

Despite the good level of concordance (83.9%) between the occurrence of N-protein specific (Roche Elecsys) and S-protein specific antibodies (in house ELISA), N-specific titers (ELECSYS) did not correlate with our in-house S-protein ELISA in the overall analysis (Figure 2). Considering this obvious discordance regarding N- and S-protein specific antibody titers, the positive population in any test with sufficient material for further testing (*n* = 161) was analyzed for neutralizing antibodies (nAbs).

**Figure 3:**
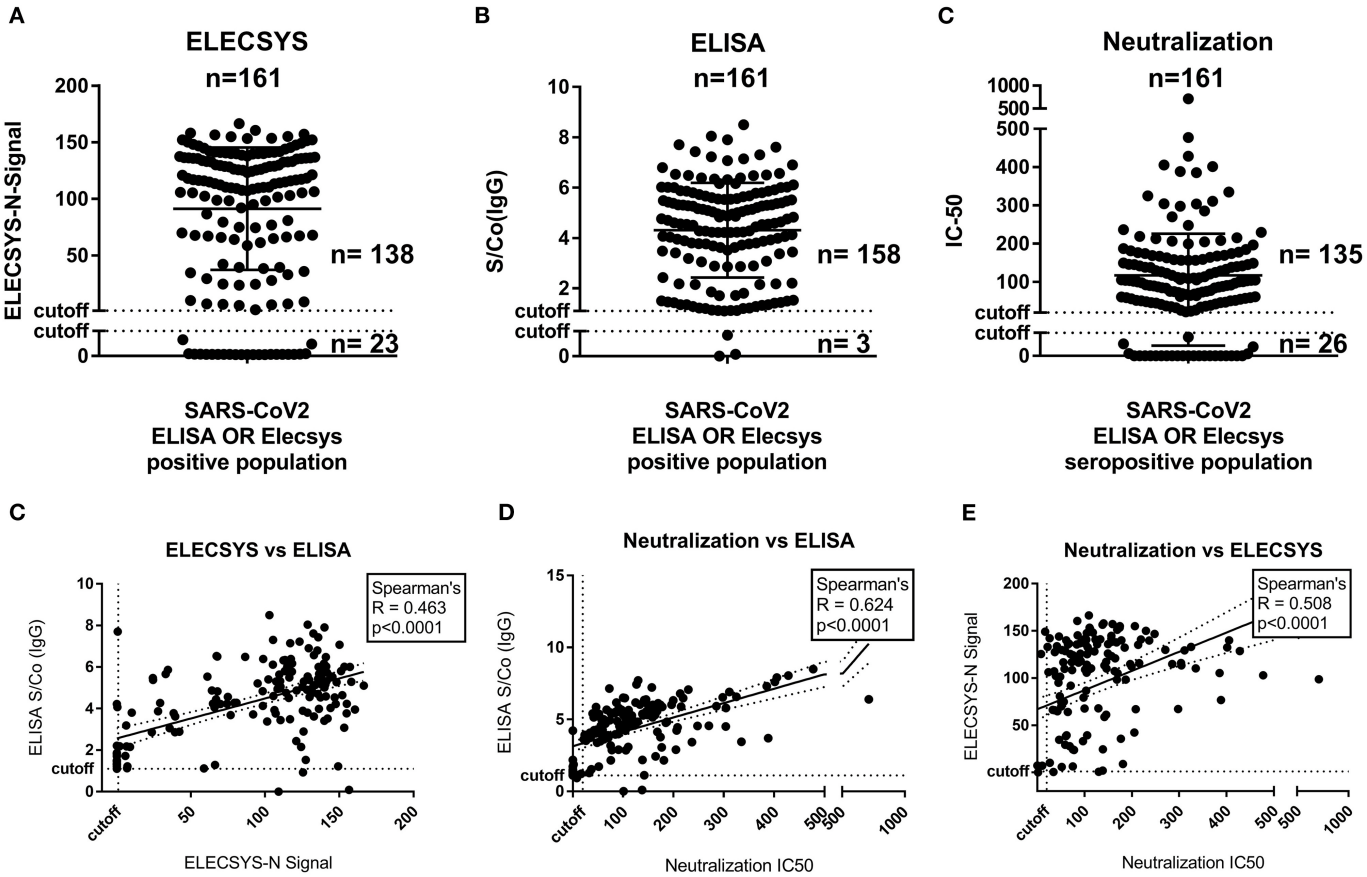
N- and S-protein specific binding antibody titer and neutralization capacity analysis of in any test positive children (*n* = 161). **(A)** Distribution of N-specific antibody signal (Elecsys, S/Co). **(B)** Distribution of S_RBD_-protein ELISA binding antibody titers (S/Co). **(C)** Distribution of Neutralization titers (IC50). **(D)** Correlation of N-specific antibody signal (Elecsys, S/Co) with S_RBD_-protein ELISA binding antibody titers (S/Co). **(E)** Correlation of N-specific antibody signal (Elecsys, S/Co) with S_RBD_-protein ELISA binding antibody titers (S/Co).

In the following neutralizing activity was detected for *n* = 135 participants, providing a total concordance of 95.7 % (*n* = 133 positive; *n* = 21 negative) and a discordance of 4.3 % (*n* = 2 N-seropositive/neutralization-negative; *n* = 5 N-seronegative/neutralization-positive) of the Elecsys result with the presence of nAbs. For comparison, the ELISA showed 83.2 % concordance (*n* = 133 positive; *n* = 1 negative) and 16.8 % discordance (*n* = 25 S-seropositive/neutralization-negative; *n* = 2 S-seronegative/Neutralization-positive) with the result of the neutralization assay (Figure 3 and Supplementary Table 1). As internal control, *n* = 81 randomly chosen negative sera (matching the age and sex distribution of the positive population) were tested for the presence of neutralizing antibodies, of which none exhibited a positive result yielding a specificity of 100% (Supplementary Figure 1).

Correlating (Spearman) the quantitative results of the three assays showed a significant correlation for each pair, while the ELISA correlated best (*R* = 0.62) with the IC-50 of the neutralization assay, the quantitative readout of the Elecsys showed inferior correlation with both the ELISA (*R* = 0.46) and the neutralization (*R* = 0.50). This was not surprising, as the manufacturer doesn't recommend any quantitative readout of the ELECSYS assay. Furthermore, no significant effects could be found on any of the three (quantitative) test results regarding age or sex of the participants (Supplementary Figure 2). Neither antibody levels nor neutralization capacity did correlate with any of the classical symptoms named in Table 2 (detailed analysis in Supplementary Figure 3).

## Discussion

In our study, performed in regions of Germany with a relatively high incidence of COVID-19 in adults in the first phase of the pandemic, approximately 6% of tested children were positive for SARS-CoV-2 antibodies in two tests directed against the N- and S-proteins of the virus. Symptoms of COVID-19 were found to be rather unspecific in children while antibody response was strong in most cases. SARS-CoV-2 neutralization capacity was independent of age, sex or symptoms in those children with antibodies and absent in those without antibodies.

This study showed an unexpected high prevalence rate of SARS-CoV-2 infections in children in Germany in the first wave, comparable to similar studies in Germany (12). The antibodies in our study were determined approximately 2 months after the peak of the first pandemic wave. Despite the closing of schools, kindergartens, and nurseries very early on in the pandemic in Germany, a surprisingly high number of children showed antibodies in our study. One possible explanation for that could be that many parents who participated in the study suspected a coronavirus infection in their children due to symptoms or outbreaks in their community. Indeed, children were explicitly not tested in the beginning of the pandemic when PCR test capacities were limited. Thus, the study may have addressed an unmet need of parents to get their children tested, which was further supported by the observation that participation in the study was overwhelming.

About 70% of the positive children showed S/Co >100 in the ELECSYS test, a value approximately representing the 97th percentile of all previously available test values (provided by Roche, personal communication). We are aware that the assay is not registered for quantitative readout, nevertheless the measures give an indication for a strong antibody response in children. Compared to the 70% of seropositive children with a mild to asymptomatic course of the initial SARS-CoV-2 infection only 21% of seropositive adults with mild symptoms showed such high values in one of our studies conducted at the same time (13). A similar observation was made for the S protein based in-house ELISA test, where also high values were observed in more than half of the positively tested children. These data may suggest that children mount stronger antibody responses to SARS-CoV-2 than adults on a regular basis.

We used two different antibody tests, one directed against the N-protein and one targeting the S-protein, which explains the slight differences and discordance in test results. With two capable antibodies used for testing at the same time, we have good confidence that we were able to catch all truly seropositive children after SARS-CoV-2 infection. Interestingly, in those few cases where children were initially positive in PCR testing, approximately 30% did not show antibody responses in our tests. This is a higher percentage than observed in our studies in adults (13). Furthermore, approximately 15% of antibody positive children showed no neutralization capacity.

Taken together, it seems that children show a somewhat dichotomous response to SARS-CoV-2 in terms of antibody generation and neutralization. While a great majority mounts exceptionally high antibody responses, a significant subgroup shows no antibodies after infection or no neutralization capacities. Both, strong-responders and non-responders, represent larger fractions of the population than in our adult study populations (13). It could be speculated that strong antibody responses may contribute to the milder acute course of the initial infection observed in children, but in adults, high levels of S-specific (and neutralizing) antibodies seem to be connected to severe courses of COVID-19 (14). On the other hand, considering the lower neutralizing antibody levels in a substantial group of children, a lower protection from reinfection is much more probable, as neutralizing antibody levels were found to be highly predictive to prevent future (symptomatic) infection (15).

Our study indicates that very few symptoms are specific for COVID-19 in children. On the other hand, only 23% of children with detectable SARS-CoV-2 antibodies were free of symptoms in the weeks before the antibody test. Interestingly, even children as young as 6 years of age were able to indicate loss of smell and taste—the only specific symptom for COVID-19 we could identify in children. It is currently debated, if a loss of smell and taste is also a feature of future mutants of SARS-CoV-2, as data for the SARS-CoV-2 delta variant suggest otherwise. Thus, screening for SARS-CoV-2 infections in children by symptoms does not seem to be useful.

A large number of children acquired antibodies against SARS-CoV-2 when family members had developed COVID-19. Therefore, we suggest that children confronted with COVID-19 in the household should systematically be screened for SARS-CoV-2 antibody responses e.g., 4 weeks after the diagnosis in the index case, thereby not missing out on potential childhood SARS-CoV-2 infections despite of mild or absent symptoms in children. Especially with new, more contagious virus variants, infections in families become even more relevant.

Based on our results we propose to screen children from households with COVID-19 cases on a regular basis for SARS-CoV-2 antibodies as well as children from areas with high prevalence of COVID-19, if any symptoms suggestive for COVID-19 occur. Alternatively, prospective PCR based test systems in schools seem to be reasonable and feasible (16). Therefore, we would recommend longitudinal antibody testing as well as vaccination; if found to be safe; for children to ensure full protection from future disease.

## Data Availability Statement

The raw data supporting the conclusions of this article will be made available by the authors, without undue reservation.

## Ethics Statement

The studies involving human participants were reviewed and approved by Ethics Committee of the University of Regensburg (file-number: 20-1865-101). Written informed consent to participate in this study was provided by the participants' legal guardian/next of kin.

## Author Contributions

OL, GL, SG, KÜ, RW, and MK were responsible for the study design. OL, GL, HB-D, SL, AS-K, DE, MH, JN, and MK performed the data collection. AT, DP, SE, PN, NB, ES-V, PK, PS, RW, and AA carried out the laboratory analysis and the data interpretation. Data Analysis was performed by DP, SE, PN, SB, RW, and MK. OL, GL, DP, SE, SB, RW, and MK wrote this manuscript. All authors contributed to the article and approved the submitted version.

## Funding

This work was supported by institutional and charity funds, RW and KÜ by the Bavarian States Ministry of Science and Arts (Grant Prospective Covid-19 Cohort Tirschenreuth, TiKoCo19). Roche Diagnostics provided Elecsys Anti-SARS-CoV-2 assay free of charge.

## The CoKiBa Study Group are (in Alphabetical Order)

Bettina Aichholzer, Georg Mair, Michaela Wruk, Imke Reischl, Paediatric Office, Dr. Aichholzer, Dr. Mair, Dr. Wruk, Reischl, Bad Endorf, Germany;

David Antos, Paediatric Office Dr. Antos, Traunstein, Germany

Stephan von Koskull, Christian Becker, Paediatric Office Dr. von Koskul, Becker, Bad Aibling, Germany

Elisabeth Beer, Hubert Schirmer, Paediatric Office Dr. Beer, Dr. Schirmer, Marktredwitz, Germany

Georg Birkinger, Paediatric Office Dr. Birkinger, Wasserburg am Inn, Germany

Andreas Blueml, Paediatric Office Blueml, Trostberg, Germany

Mona Castrop, Paediatric Office Dr. Castrop, Regensburg, Germany

Jost Dieckerhoff, Paediatric Office Dr. Dieckerhoff, Rosenheim, Germany

Renate Eichhorn, Paediatric Office Dr. Eichhorn, Regensburg, Germany

Dominik Ewald, Paediatric Office Dr. Heuschmann & Dr. Ewald, Regensburg/Regenstauf, Germany

Gudrun Fleck, Alfred Heihoff, Paediatric Office Dr. Fleck, Dr. Heihoff, Regensburg, Germany

Jürgen Geuder, Paediatric Office Dr. Geuder, Freilassing, Germany

Jens Grombach, Paediatric Office Dr. Grombach, Neuoetting, Germany

Peter Gutdeutsch, Florian Segerer, Paediatric Office Dr. Gutdeutsch, Dr. Segerer, Regensburg, Germany

Thomas Habash, Sonja Habash, Paediatric Office Dres. Habash, Cham, Germany

Susanne Harner, University Childrens‘Hospital Regensburg (KUNO) at the Hospital St. Hedwig of the Order of St. John University of Regensburg, Regensburg, Germany

Christoph Herbst, Paediatric Office Dr. Herbst, Edling, Germany

Daniela Heuschmann, Paediatric Office Dr. Heuschmann & Dr. Ewald, Regensburg/Regenstauf, Germany

Meike Hofmann, Paediatric Office Hofmann, Mitterteich, Germany

Michael Horn, Paediatric Office Dr. Horn, Schoenau am Chiemsee, Germany

Birgit Jork-Kaeferlein, Monika Schwarz, Reinhard Hopfner, Paediatric Office Dr. Jork-Kaeferlein, Dr. Schwarz, Dr. Hopfner, Prien, Germany

Guido Judex, Bastian Baumgartner, Monika Corbacioglu, Sabrina Lindner, Bettina Meinel, Alena Bauer, Hannes Löw, Annamaria Szulagyi-Kovacs, Paediatric Office Dr. Judex, Dr. Baumgartner, Dr. Corbacioglu, Dr. Lindner, Dr. Meinel, Bauer, Löw, Szulagyi-Kovacs, Regensburg, Germany

Michael Kabesch, University Childrens‘Hospital Regensburg (KUNO) at the Hospital St. Hedwig of the Order of St. John University of Regensburg, Regensburg, Germany

Annegret Klein, Paediatric Office Dr. Klein, Oberaudorf, Germany

Cosima Koering, Paediatric Office Dr. Koering, Altoetting, Germany

Niclas Landvogt, Claudia Soehngen, Karin Rasp, Gudrun Schick-Niedermeier, Paediatric Office Dr. Landvogt, Dr. Soehngen, Traunreut, Germany

Marinus Laub, Paediatric Office Laub, Rosenheim, Germany

Otto Laub, Paediatric Office Laub, Rosenheim, Germany

Georg Leipold, Petra Schmid-Seibold, Paediatric Office Dr. Leipold Schmid-Seibold, Regensburg, Germany

Johannes Pawlak, Michaela Reitz, Paediatric Office Dr. Pawlak, Dr. Reitz, Rosenheim, Germany

Georg Puchner, Paediatric Office Dr. Puchner, Regensburg, Germany

Christiane Razeghi, Stefan Razeghi, Paediatric Office Dres. Razeghi, Miesbach, Germany

Christine Rehe, Klaus Rehe, Paediatric Office Dres. Rehe, Kolbermoor, Germany

Matthias Scheffel, Ludwig Kaesbauer, Paediatric Office Dr. Scheffel, Dr. Kaesbauer, Muehldorf, Germany

Roland Schmid, Michael Strobelt, Paediatric Office Dr. Schmid, Dr. Strobelt, Bruckmuehl, Germany

Nina Schoetzau, Paediatric Office Dr. Schoetzau, Miesbach, Germany

Marko Senjor, Paediatric Office Dr. Senjor, Wasserburg, Germany

Michael Sperlich, Paediatric Office Dr. Sperlich, Ampfing, Germany

Guenter Theuerer, Guenter Steidle, Paediatric Office Dr. Theurer, Dr. Steidle, Traunstein, Germany

German Tretter, Paediatric Office Dr. Tretter, Altenstadt an der Waldnaab, Germany

Victor von Arnim, Paediatric Office Dr. von Arnim, Roding, Germany

Marlene Volz-Fleckenstein, Paediatric Office Dr. Volz-Fleckenstein, Regensburg, Germany

## Conflict of Interest

JN was employed by the company Maganamed Limited. The remaining authors declare that the research was conducted in the absence of any commercial or financial relationships that could be construed as a potential conflict of interest.

## Publisher's Note

All claims expressed in this article are solely those of the authors and do not necessarily represent those of their affiliated organizations, or those of the publisher, the editors and the reviewers. Any product that may be evaluated in this article, or claim that may be made by its manufacturer, is not guaranteed or endorsed by the publisher.
