# Supplementary material for: Symptoms, SARS-CoV-2 Antibodies, and Neutralization Capacity in a Cross Sectional-Population of German Children
Source: Front Pediatr. 2021 Oct 4;9:678937. doi: 10.3389/fped.2021.678937 (PMC8522552; doi:10.3389/fped.2021.678937)

**SUPPLEMENTARY MATERIAL**

**Supplementary table 1:** Comparative analysis of three serological test results (Cutoffs: Roche ELECSYS N S/Co≥1; S_RBD_-ELISA S/Co≥1.1; Neutralization IC_50_≥20) of the seropositive population in any test (n=161) visualizing the positive, negative as well as concordant and discordant results.

| ELECSYS vs Neutralization | | | ELISA vs Neutralization | | | ELECSYS vs ELISA | | |
| --- | --- | --- | --- | --- | --- | --- | --- | --- |
|  | Neutralization | |  | Neutralization | |  | ELISA | |
| Elecsys | positive | negative | ELISA | positive | negative | ELECSYS | positive | negative |
| positive  #; % | 133; 82.6 | 5;  3.1 | positive  #; % | 133; 82.6 | 25;  15.5 | positive  #; % | 135; 83.9 | 3;  1.9 |
| negative  #; % | 2;  1.2 | 21;  13.0 | negative  #; % | 2;  1.2 | 1; 0.6 | negative  #; % | 23;  14.3 | 0;  0 |
| total concordance  % | **95.7** | | **total concordance**  **%** | **83.2** | | **total concordance**  **%** | **83.9** | |
| total discordance  % | **4.3** | | **total discordance**  **%** | **16.8** | | **total discordance**  **%** | **16.1** | |

**Supplemental Figure 1** Age and sex distribution of **A** the seropositive population in any test (n=161), **B** the negative control (n=81; randomly picked based on age and sex of the pos. population) and **C** the neutralization test result of the negative control (n= 81, all negative)

**
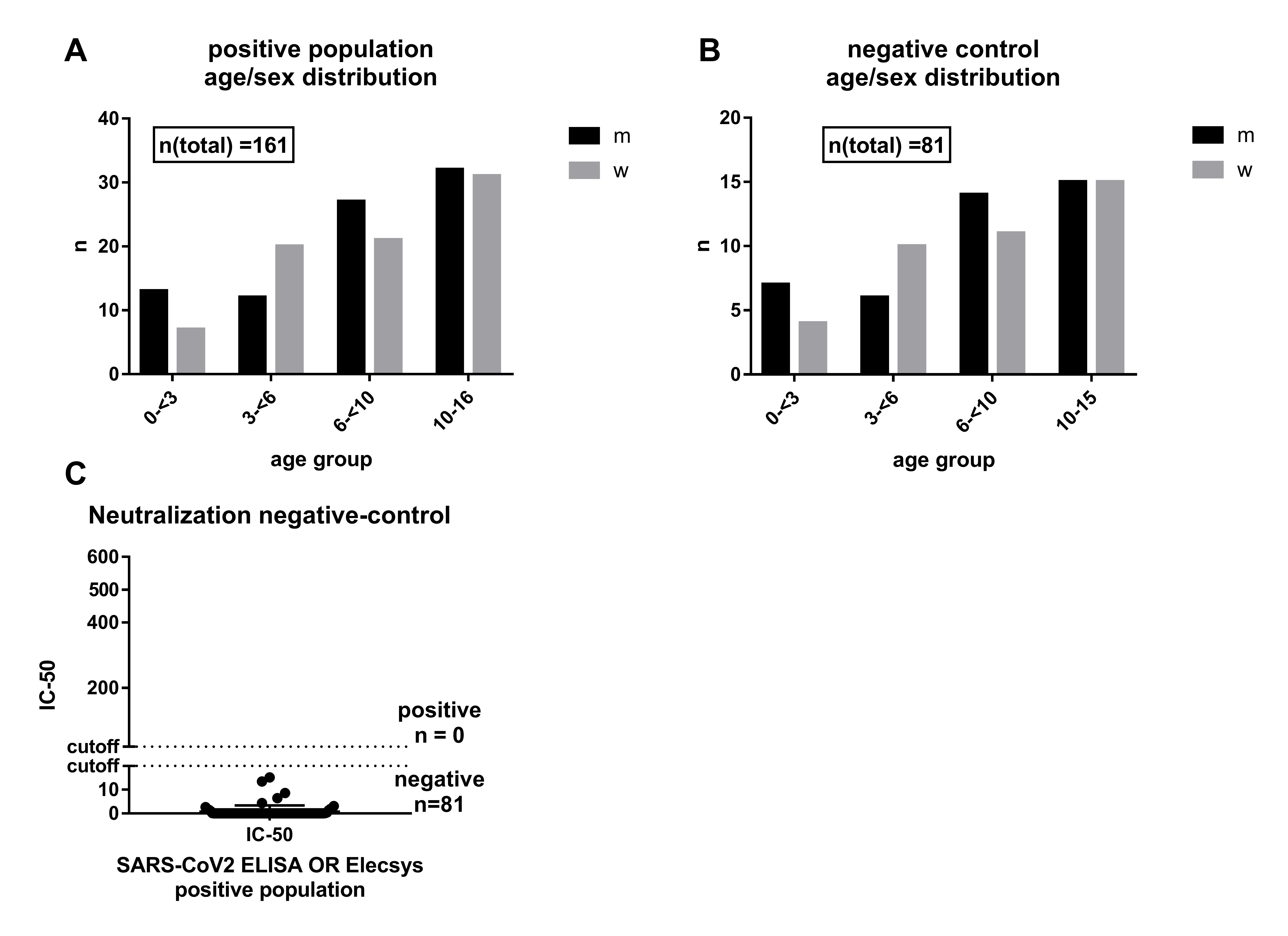
**

**Supplemental Figure 2** Spearman Correlation of **A** ELECSYS N (S/Co; n.s.; R= -0.05), **B** Neutralization (IC_50_; n.s.; R=-0.11) and **C** S_RBD_-ELISA (S/Co; n.s.; R=-0.32) with the age of the participants. And comparison of the male and female population (unpaired t-test) for **D** Neutralization (IC_50_; n.s.), **E** S_RBD_-ELISA (S/Co; n.s.) and **F** ELECSYS N (S/Co; n.s.)


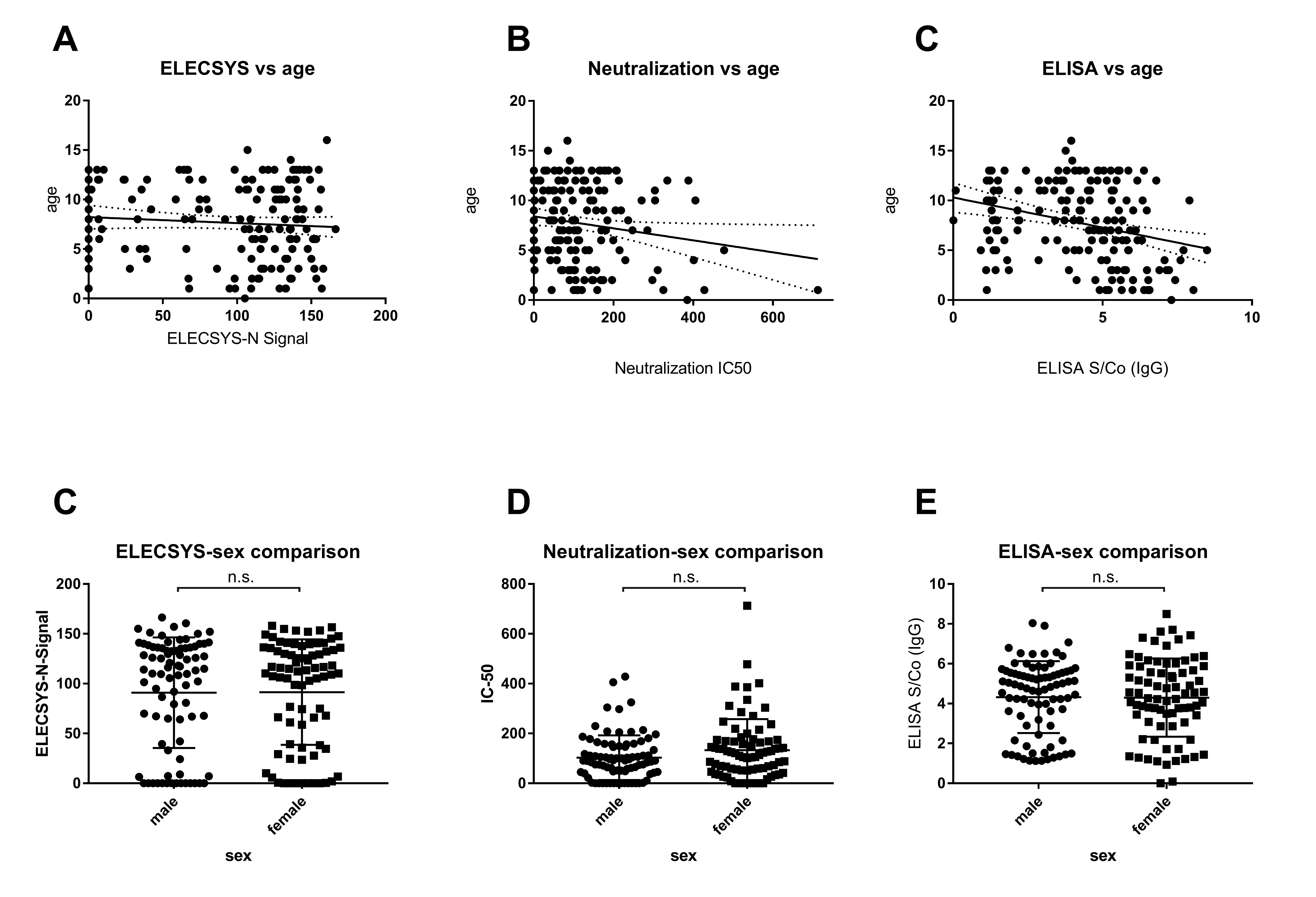


**Supplemental Figure 3** Association of Antibody Values, Neutralization Capacity and the Antibody to Neutralization Ratio with Classical Symptoms of COVID19

Legend: AB value (ELECSYS), neutralization capacity and a combined measure of AB value (ELECSYS) and neutralization capacity (ELECSYS >130, neutralization capacity <50) are presented in columns, selected SARS-CoV-2 infection related symptoms are presented in rows. All AB positive children with questionnaire data on symptoms were included in the analyses (N=161/162). T-tests and chi²-tests revealed no statistically significant difference in any of the displayed comparisons.


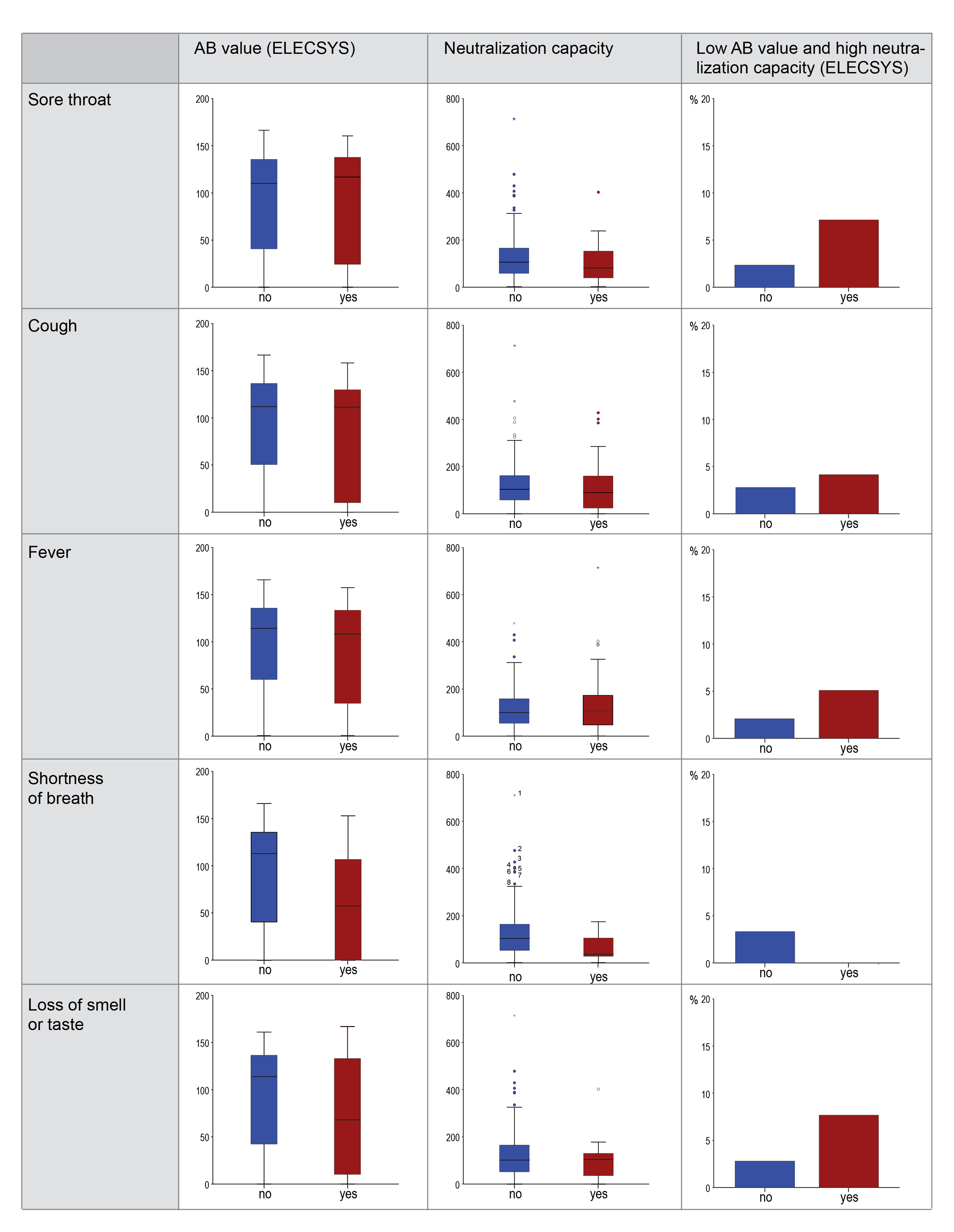

Supplement: Supplementary file 1 [file Data_Sheet_1.docx]
